# A novel defined risk signature based on pyroptosis-related genes can predict the prognosis of prostate cancer

**DOI:** 10.1186/s12920-022-01172-5

**Published:** 2022-02-08

**Authors:** Ding Hu, Qingfei Cao, Ming Tong, Chundong Ji, Zizhi Li, Weichao Huang, Yanyang Jin, Guangquan Tong, Yutao Wang, Pengfei Li, Huashan Zhang

**Affiliations:** 1 Department of Urology, Jinzhou Medical University, The First Hospital of Jinzhou Medical University, Jinzhou, Liaoning China; 2 Department of Urology, Affiliated Hospital of Panzhihua University, Panzhihua, Sichuan China; 3 Department of Urology, China Medical University, The First Hospital of China Medical University, Shenyang, Liaoning China

**Keywords:** Pyroptosis, TCGA, GEO, Prostate cancer, Prognostic signature, Immune infiltration

## Abstract

**Background:**

Pyroptosis can not only inhibit the occurrence and development of tumors but also develop a microenvironment conducive to cancer growth. However, pyroptosis research in prostate cancer (PCa) has rarely been reported.

**Methods:**

The expression profile and corresponding clinical data were obtained from The Cancer Genome Atlas (TCGA) and Gene Expression Omnibus (GEO) databases. Patients were divided into different clusters using consensus clustering analysis, and differential genes were obtained. We developed and validated a prognostic biomarker for biochemical recurrence (BCR) of PCa using univariate Cox analysis, Lasso-Cox analysis, Kaplan–Meier (K–M) survival analysis, and time-dependent receiver operating characteristics (ROC) curves.

**Results:**

The expression levels of most pyroptosis-related genes (PRGs) are different not only between normal and tumor tissues but also between different clusters. Cluster 2 patients have a better prognosis than cluster 1 patients, and there are significant differences in immune cell content and biological pathway between them. Based on the classification of different clusters, we constructed an eight genes signature that can independently predict the progression-free survival (PFS) rate of a patient, and this signature was validated using a GEO data set (GSE70769). Finally, we established a nomogram model with good accuracy.

**Conclusions:**

In this study, PRGs were used as the starting point and based on the expression profile and clinical data, a prognostic signature with a high predictive value for biochemical recurrence (BCR) following radical prostatectomy (RP) was finally constructed, and the relationship between pyroptosis, immune microenvironment, and PCa was explored, providing important clues for future research on pyroptosis and immunity.

**Supplementary Information:**

The online version contains supplementary material available at 10.1186/s12920-022-01172-5.

## Background

Prostate cancer (PCa) is the most common malignant tumor in male genitourinary system. According to Cancer Statistics, there were about 248,530 new cases and 34,130 deaths in the United States in 2021, accounting for 26% of the total incidence of malignant tumors and 11% of the total mortality [1]. Most patients with localized cancers receive standard treatments, such as radical prostatectomy (RP) or radiation therapy [2]. However, biochemical recurrence (BCR) occurs in approximately 20–30% of patients [3]. BCR patients develop clinical relapses and metastases that ultimately result in death. While numerous indicators exist for predicting the prognosis of PCa patients, such as Gleason score and prostate-specific antigen (PSA) [4, 5], their ability to predict the BCR time of patients is limited. Therefore, developing a biomarker with high accuracy and strong specificity is critical for predicting the prognosis and guiding PCa patients’ treatment. Pyroptosis, also known as cellular inflammatory necrosis, is a programmed cell death that manifests as constantly enlarging cells, releasing cell contents and thus activating a strong inflammatory response [6]. Unlike apoptosis, pyroptosis requires inflammasomes and gasdermin family to act as executors [7]. Pyroptosis is a caspase dependent regulated cell death, and it is driven primarily by the pore forming proteins gasdermin D (GSDMD) or gasdermin E (GSDME / DFNA5) [8]. During stresses such as inflammasome activation, GSDMD can be cleaved by CASP1, CASP11, or CASP8 to generate the N-terminal fragment of GSDMD (GSDMD-N) [9]. In contrast, the production of GSDME-N is mediated by CASP3 [10]. After oligomerization, GSDMD-N or GSDME-N forms pores in the plasma membrane, leading to pyroptotic cell death. Pyroptosis is believed to play a dual action in tumorigenesis by inhibiting the occurrence and development of tumors and developing a microenvironment that provides cancer with nutrients and accelerates its growth [11]. At present, numerous studies have revealed that pyroptosis is critical for tumor cell proliferation, invasion, and metastasis. For instance, transcription factor p53 inhibits tumor growth by promoting pyroptosis in non-small cell lung cancer [12]. In gastric cancer, a new pyroptosis-related gene signature has been identified to predict prognosis [13]. Nevertheless, the prognostic value of genes associated with pyroptosis has not been explored in PCa patients.

This study used bioinformatics methods to investigate the expression level, clinical value, and related immune process of pyroptosis-related genes (PRGs) in PCa patients to develop a good model for predicting the PCa patients' prognosis and improving the treatment effect of disease.

## Methods

### Data collection and collation

**Fig. 1:**
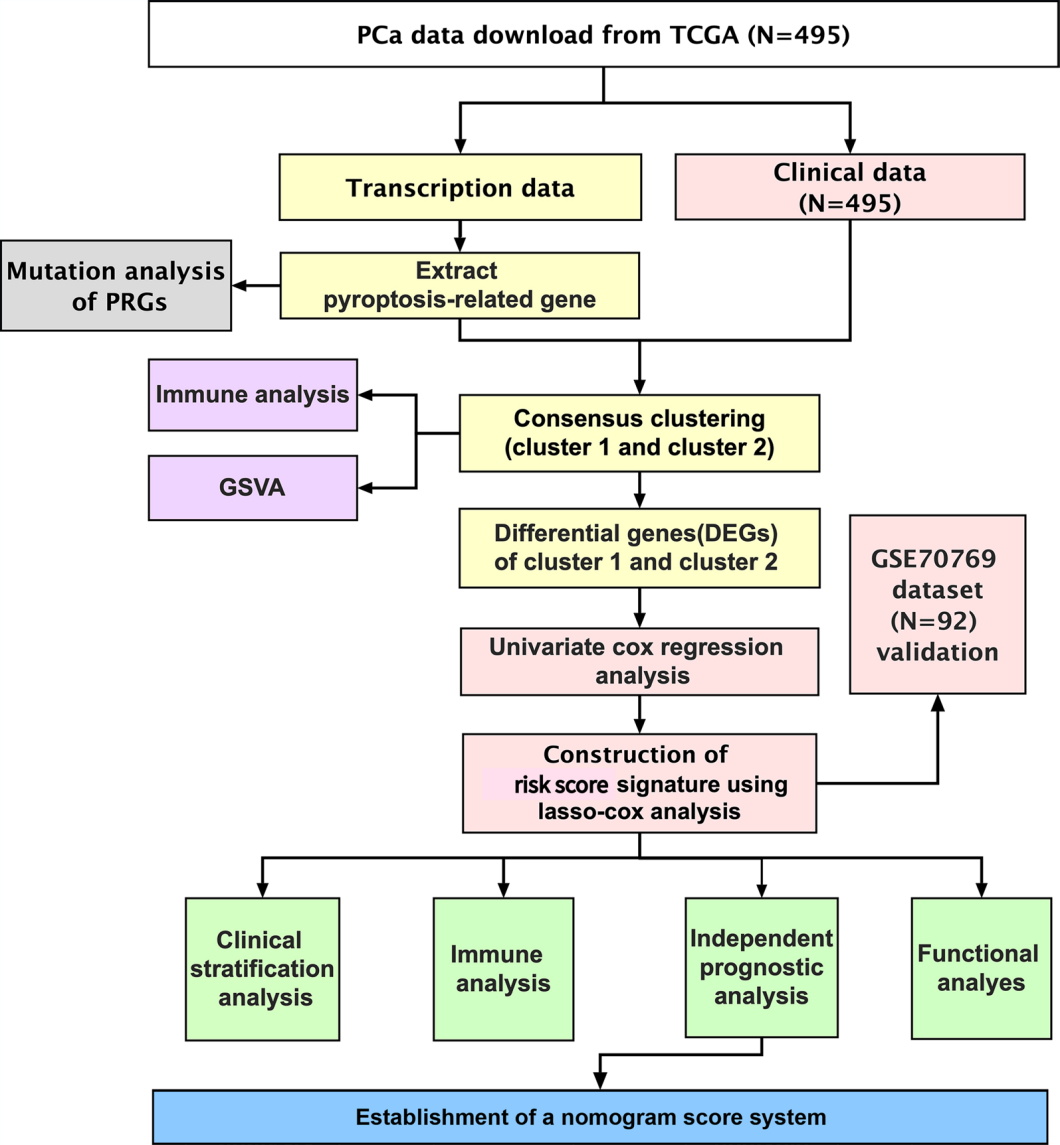
The workflow chart of this research

The workflow of this study is depicted in Fig. 1. We obtained transcriptome and clinical data of 495 prostate cancer patients from the Cancer Genome Atlas (TCGA) database (https://portal.gdc.cancer.gov). Corresponding data from 92 patients in the Gene Expression Omnibus (GEO) database (https://www.ncbi.nlm.nih.gov/geo/) (GSE70769) were used for further model validation. In addition, mutation and copy number variation (CNV) data were also obtained from TCGA database. The batch effect of non-biotech deviations was removed using R package "SVA"[14]. We obtained 52 PRGs from previous literature as well as from the REACTOME_ PYROPTOSIS gene set in the Molecular Signatures Database (MSigDB, https://www.gsea-msigdb.org/gsea/index.jsp) [15–18] (Additional file 7: Table S1). We explored the imbalance of their expression in tumor tissues using R-package "limma"[19] and plotted the heatmap using R-package "heatmap". A protein–protein interaction (PPI) network for the PRGs was constructed with Search Tool for the Retrieval of Interacting Genes (STRING, https://string-db.org/).

### Mutation analysis of PRGs

The landscape of mutations of 52 PRGs was represented by the waterfall diagram drawn by R-package "maltools", and CNV position changes of 52 PRGs on 23 chromosomes were drawn using R-package "RCircos".

### Consensus clustering

Unsupervised clustering analysis was performed using R-package "Consensus Cluster Plus" and the "k-means" method to identify different modification patterns according to the expression of these PGRs and classify the patients for further analysis [20]. These steps were repeated 1000 times to ensure the classification's stability.

### Immune microenvironment analysis

The algorithms "CIBERSORT [21]" and "ESTIMATE [22]" were used to quantify immunocytes and evaluate the purity of tumors from different clusters. Single sample gene set enrichment analysis (ssGSEA) [23] was employed to quantify immune cells, immune function, and pathways between different risk score subgroups.

### Gene set variation analysis (GSVA)

We downloaded "c2.cp.kegg.v7.2.symbols" from MSigDB database and performed GSVA analysis on different clusters using R package "GSVA"[24], where |log2FC| > 0.2 and adjusted *p* < 0.05 were considered significantly enriched and presented as a heatmap.

### Differentially expressed genes (DEGs)

The R-package "limma"[19] was used to obtain the differential gene expression between different clusters, with the filtering criteria of |log2FC| > 1 and false discovery rate (FDR) < 0.05.

### Univariate and multivariate Cox regression analyses

Univariate Cox regression analysis was used to screen for prognosis-related DEGs. In addition, univariate and multivariate Cox analyses were also employed to test independent prognostic performance of signature. *P* < 0.05 was considered significant.

### Establishment of a risk signature based on PRGs cluster

Using TCGA and GSE70769 cohorts as training and testing sets, respectively, the most useful predictive features from the training set were obtained using progression-free survival (PFS) by Lasso-Cox analysis of prognostic DEGs and were validated in the testing set. The risk score for each patient is calculated as follows:

$$risk score={\sum }_{i=1}^{n}coef(i)*expr(i)$$

where n, coef(i), and expr(i) respectively represent the number, corresponding coefficient, and corresponding expression of signature genes. TCGA dataset and GSE70769 dataset of patients were each assigned to high-and low-score groups by median risk score of TCGA dataset. We evaluated the signature's ability to differentiate between subgroups of patients using Kaplan–Meier survival curve and determined the model's accuracy using time-dependent receiver operating characteristics (ROC) curves.

### Functional enrichment analysis of DEGs between low- and high-score groups

PCa patients in TCGA cohort were divided into two subgroups based on median risk score. DEGs between high- and low-score groups were screened based on filtration criteria (|log2FC| ≥ 0.585 and FDR < 0.05). These DEGs were analyzed for Gene Ontology (GO) and Kyoto Encyclopedia of Genes and Genomes (KEGG) using R-package “clusterProfiler”.

### Construction of a nomogram and performance detection

A nomogram model was constructed using R-package “rms” based on risk score and other independent clinical factors to enhance the clinical utility of risk signature. Calibration charts were used to verify the nomogram's accuracy.

### Statistical analysis

All visualization and statistical analyses for this study were performed using R version 4.1.0 and the corresponding feature packages. Differences between groups for different data sets or different classifications of data were determined using chi-square tests. Comparisons between two groups were performed using Wilcoxon test. In addition, Kaplan Meier (K–M) survival analysis was conducted using a log-rank test. The above statistical methods were considered statistically significant when *p* < 0.05.

## Results

### Defining of the expression of PRGs in PCa

**Fig. 2:**
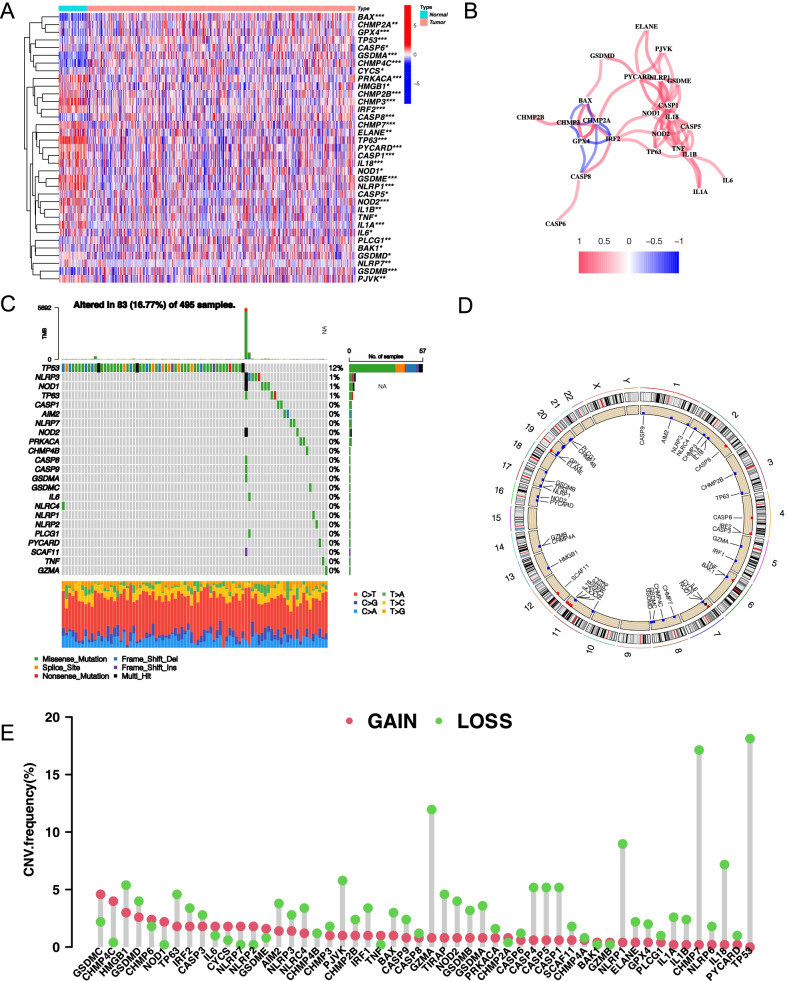
Characterization of pyroptosis-related genes at the biological level in tumor. **A** The heatmap revealed that expression levels of 35 of 52 PRGs in tumor and normal tissues were imbalanced, with red representing high expression and blue representing low expression (Wilcoxon test, *P < 0.05; **P < 0.01; ***P < 0.001). **B** Correlation network of 35 PRGs differentially expressed between cancer and normal tissues (correlation coefficient > 0.4, with red line representing positive correlation and blue line representing negative correlation). **C** Genomic changes from 495 PCa samples from TCGA with waterfalls representing information on different PRGs mutations. A note at the bottom of corresponding color indicates different mutation types. The histogram above represents the tumor mutation burden for each sample. The number on the right indicates the mutation frequency. **D** The location of PRGs at which CNV occurs on chromosome. **E** The frequency of copy number variation (CNV) for different PRGs indicated that deletion with copy number existed in most PRGs

The prostate cancer expression profile in the TCGA database included 52 normal samples and 495 tumor samples. First, as pyroptosis can promote tumor proliferation and invasion, we used the TCGA database to analyze the differences of the expression levels of these 52 PRGs in tumor tissue and normal tissue, Gleason Score ≤ 7 group and Gleason Score > 7 group, respectively. The results indicated a difference in the expression levels of 35 PRGs between PCa and precancerous tissues and difference in the expression levels of 22 PRGs between Gleason score ≤ 7 group and Gleason score > 7 group (Fig. 2A; Additional file 8: Table S2, Wilcoxon test, *P < 0.05; **P < 0.01; ***P < 0.001). The expression levels of IL1A, TP63, ELANE, IL6, CASP1, GSDME, NLRP1, IL18, NOD2, PYCARD, IL1B, NLRP7, CHMP3, IRF2, PRKACA, TNF, CHMP7, CASP5, CHMP2B, PJVK, NOD1, HMGB1, and GSDMD were down-regulated in tumor tissues, whereas those of BAK1, CASP6, CYCS, PLCG1, TP53, CHMP2A, CASP8, GPX4, BAX, CHMP4C, GSDMB, and GSDMA were down-regulated in normal tissues. PPI network was constructed by string (Additional file 1: Figure S1A), and hub genes in it were identified using "MCODE" plugin of cytoscape software, which were CASP5, GSDMD, IL18, CASP1, TNF, IL1A, IL6, IL1B, NLRP1, and PYCARD (Additional file 1: Figure S1B). The correlation network consisting of 35 PRGs is depicted in Fig. 2B (correlation coefficient > 0.4, positive correlation is shown with red line, negative correlation is shown with blue line.

### Landscape of genetic variation of PRGs in PCa

We analyzed the incidence of somatic and copy number mutations in 52 PRGs from PCa. As illustrated in Fig. 2C, 83 of 495 (16.77%) PCa samples had genetic mutations. 23 of 52 PRGs were mutated, with TP53 showing the highest mutation frequency. Among these types of gene mutations, missense mutations are the most frequent. We also found that at the CNVs level, most of the focus was on missing copies (Fig. 2E). Additionally, we identified changes in regulatory factors with CNV features on chromosomes (Fig. 2D).

### Identification of 52 PRGs-mediated PCa classification patterns

**Fig. 3:**
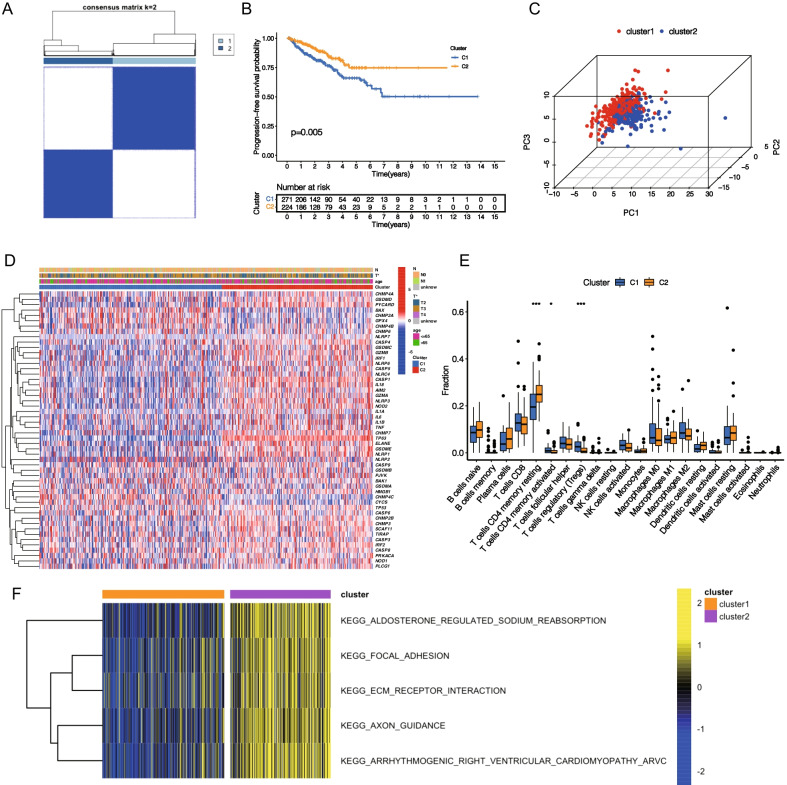
Subunit types based on PRGs in PCa and their immune microenvironment. **A** Consensus clustering matrix for k = 2. **B** The results of K-M analysis indicated that cluster 1 had a significantly lower tumor progression-free survival than cluster 2 (log-rank test, *p* = 0.005). **C** Principal component analysis (PCA) showed that these PRGs could well divide TCGA cohort into two distinct clusters. **D** Heatmap with different clinical features revealed that PRGs had differential expression in different clusters. **E** Immune cell infiltration of different clusters based on "CIBERSORT" algorithm (Wilcoxon test, *P < 0.05; **P < 0.01; ***P < 0.001). **F** The heatmap shows the biological pathway to pyroptosis-related clusters by gene set variation analysis (GSVA) (|log2FC|> 0.2 and adjusted *p* < 0.05)

Based on the expression levels of 52 PRGs, two different regulatory patterns, cluster 1 (n = 271) and cluster 2 (n = 224) were identified using unsupervised clustering (Fig. 3A; Additional file 9: Table S3). Following that, the principal component analysis (PCA) confirmed that cluster 1 and cluster 2 could be distinguished using 52 PRGs (Fig. 3C). We found that the expression levels of these PRGs were significantly different between the two clusters (Fig. 3D). Simultaneously, cluster 2 had a significantly higher PFS rate than cluster 1 (Fig. 3B) (log-rank test, *p* = 0.005). To explore the differences in the immune microenvironment between these two patterns, we performed immunocyte infiltration analysis and tumor purity evaluation using “CIBERSORT” and “ESTIMATE” algorithms (Fig. 3E; Additional file 2: Figure S2A–D). The results indicated that the contents of Tregs and CD4+ activated memory T cells in cluster 1 were significantly increased, whereas the content of resting memory CD4 T cells was significantly decreased. Meanwhile, the tumor purity of cluster 1 was higher. PD-1, PD-L1, PD-L2, and CTLA4 are common immune checkpoints. We quantified immune checkpoints of different clusters, and the results indicated that the content of these immune checkpoints was higher in cluster 2 (Additional file 3: Figure S3). To explore the difference in biological behavior between these two clusters, we performed a GSVA enrichment analysis (Fig. 3F; Additional file 10: Table S4). The results showed that cluster 2 was mainly enriched in carcinogen pathways, such as focal adhesion, EMC receptor interaction, etc.

### Construction and verification of risk signature based on PRG clusters

**Fig. 4:**
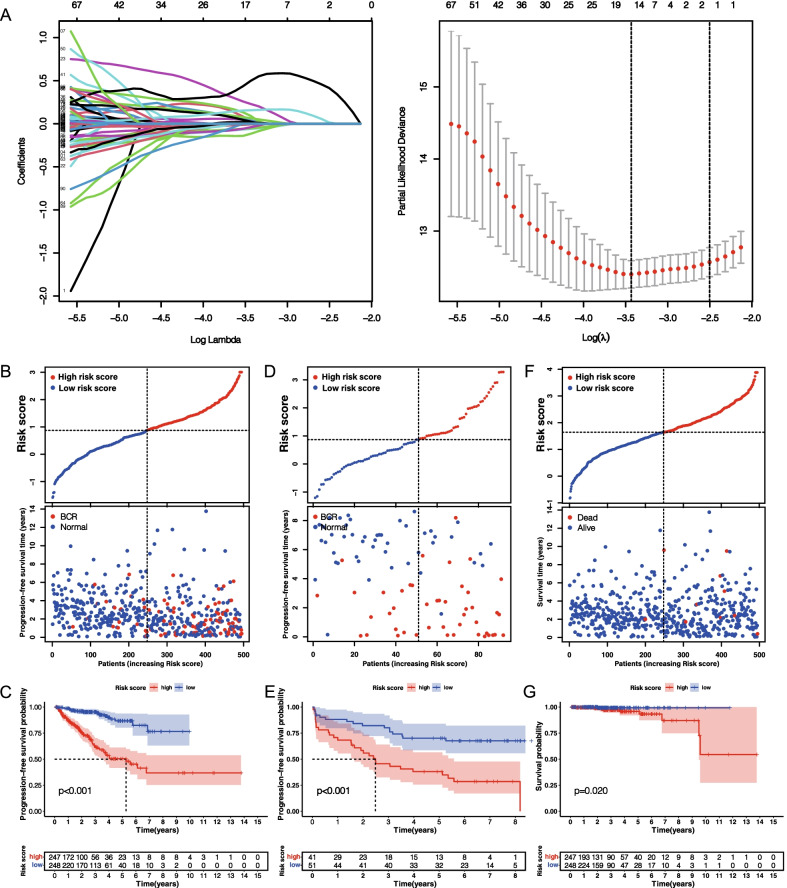
Eight genes-based prognostic signature was constructed using Lasso-Cox regression analysis, and its efficacy was tested. **A** The distribution of partial likelihood deviation of Lasso coefficient preserves 15 variables when partial likelihood deviation reaches the minimum (Log Lambda = − 3.45). PFS risk profile for risk score-based patients in TCGA cohort (increased number of patients with biochemical relapse (BCR) as scores increased) (**B**), K–M surviving curve (log-rank test, *p* < 0.001) (**C**). PFS risk profile for risk score-based patients in GSE70769 cohort (same as TCGA cohort, with increased incidence of BCR as scores increased) (**D**), K–M survival curve (log-rank test, *p* < 0.001) (**E**). Overall survival risk profile for risk score-based patients in TCGA cohort (increased number of patients die as the score increases) (**F**), K–M survivable curve (log-rank test, *p* = 0.02) (**G**)

**Fig. 5:**
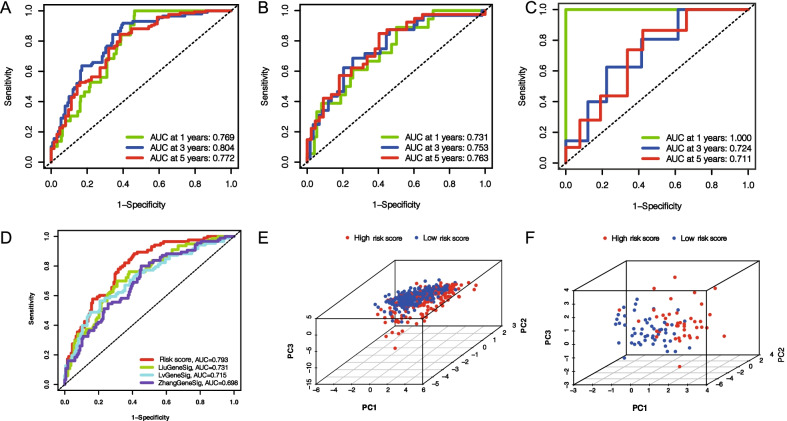
The signature has high accuracy and applicability. 1, 3, 5-year ROC curve for risk score-based patients in TCGA cohort (**A**) and GSE70769 cohort (**B**). **C** 1, 3, 5-year ROC curve of overall survival risk for risk score-based patients in TCGA cohort. **D** The ROC curve for the 3-year progression-free survival prediction of risk signature and the other four prognostic signatures. Results from PCA in TCGA cohort (**E**) and GSE70769 cohort (**F**) showed that risk score well divided the samples into two subgroups

To better apply these subtypes to the clinical treatment of PCa and determine the specific score for each patient, we next explored the differences between the two patterns and identified specific gene signatures. Additionally, we quantified gene signatures for use in predicting prognosis of individual patients. First, using |log2FC|> 1 and FDR < 0.05 as the filtering criteria, we identified 516 DEGs according to the above two patterns (Additional file 11: Table S5). Following that, according to univariate Cox regression analysis results, the obtained 110 prognosis-related genes were used as candidate molecules for constructing a prognosis signature (*p* < 0.05) (Additional file 4: Figure S4; Additional file 12: Table S6). Through Lasso-Cox analysis, a signature consisting of eight genes was finally obtained (Fig. 4A; Table 1). By the product of the expression level of each gene and its coefficient, the risk score of each sample can be obtained, and risk score = (0.741 * expression CENPA) + (− 0.134 * expression LCN2) + (0.802 * expression COL7A1) + (0.222 * expression ALB) + (− 0.610 * expression UBXN10) + (0.302 * expression SPZ1) + (− 0.227 * expression SCNN1A) + (− 0.111 + expression TFF3). Based on the median risk score (0.873), all patients were assigned to high- and low-score groups (Additional file 13: Table S7). We can find that BCR patients gradually increased as the score increases (Fig. 4B). PFS rates were significantly lower in the high-score group than in the low-score group, as determined by K–M survival analysis (log-rank test, *p* < 0.001) (Fig. 4C). ROC analysis revealed AUC values of 0.769, 0.804, and 0.772 for 1, 3, and 5 years, respectively, indicating high signature accuracy (Fig. 5A). To further verify the accuracy of signature, validation was performed using GSE70769 cohort from GEO database (GSE70769 cohort was divided into high and low scoring groups using the median of TCGA cohort) as shown in Fig. 4D, E and Additional file 14: Table S8. The two groups had significantly different prognoses (log-rank test, *p* < 0.001) and AUC values of 0.731, 0.753, and 0.763 at 1, 3, and 5 years respectively (Fig. 5B), further demonstrating signature accuracy. Furthermore, we were surprised to identify that this signature reflected the overall survival (OS) rate of PCa patients (log-rank test, *p* = 0.02) (Fig. 4F, G), and AUC values of 1, 0.724, and 0.711 at 1, 3, and 5 years respectively (Fig. 5C). Researchers have studied prognosis models of PCa with modification conditions such as ferroptosis, m6A, and immune score, such as seven-gene signature discovered by Liu et al. [25], eleven-gene signature discovered by Zhang et al. [26], and seven-gene signature discovered by Lv et al. [27]. Through comparison, we found that the accuracy of our risk signature was superior to other prognostic models according to AUC values of the ROC curve (Fig. 5D). Subsequently, we confirmed by PCA that risk score could be used as an independent indicator to distinguish PCa patients (Fig. 5E, F).

**Table 1:** Results of multivariate Cox proportional hazard regression analysis of candidate genes in progression-free survival rate

Gene	Coefficient
CENPA	0.741
LCN2	− 0.134
COL7A1	0.802
ALB	0.222
UBXN10	− 0.610
SPZ1	0.302
SCNN1A	− 0.227
TFF3	− 0.111

Coefficient: Weights for each gene obtained by multivariate Cox analysis.

### Risk signature has excellent independent prognostic value

**Fig. 6:**
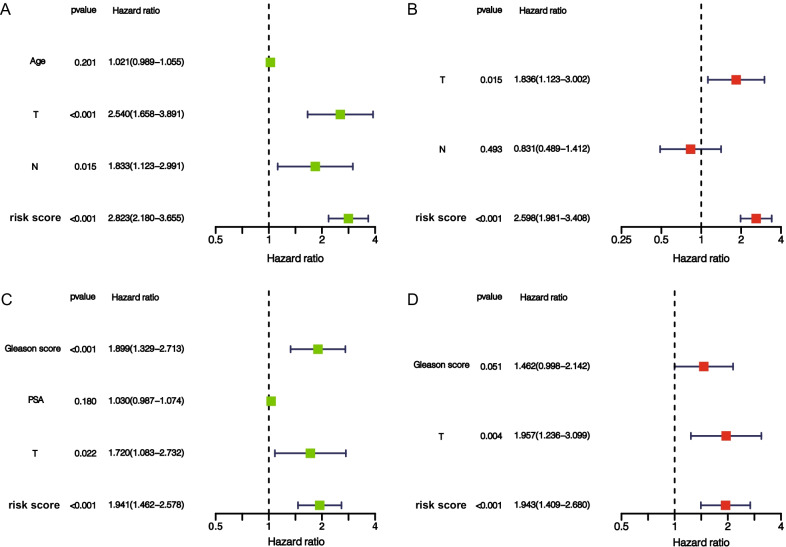
Risk score is an excellent independent prognostic factor in PCa patients. Forest map based on univariate Cox analysis (**A**) and multivariate Cox analysis (**B**) of TCGA cohort. Forest map based on univariate (**C**) and multivariate (**D**) Cox analyses of GSE70769 cohort

**Fig. 7:**
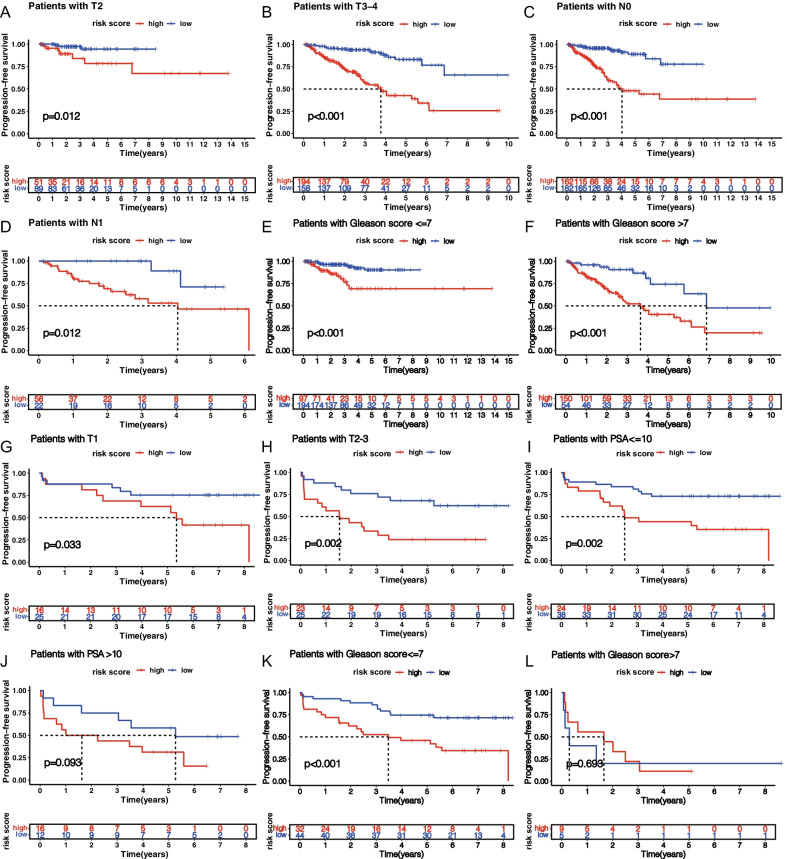
Risk score based clinical stratification in TCGA and GSE70769 sets. Kaplan–Meier survival curves for patients with T2 (**A**), T3–4 (**B**), N0 (**C**), N1 (**D**), Gleason score ≤ 7 (**E**), Gleason score > 7 (**F**) in TCGA set, and T1 (**G**), T2–3 (**H**), PSA ≤ 10 (**I**), PSA > 10 (**J**), Gleason score ≤ 7 (**K**), Gleason score > 7 (**L**) in GSE70769 set (log-rank test)

To determine whether signature could represent its prognostic value independently of other clinical factors such as T-stage, we performed univariate and multivariate Cox analyses based on TCGA and GEO cohorts, respectively (Fig. 6A–D). The results revealed that risk score and T-stage were independent factors affecting PCa patients' prognosis, and the hazard ratio (HR) of risk score in TCGA and GEO cohorts was 2.598 and 1.943, respectively. The heatmap containing clinical features demonstrates that signature can significantly distinguish the patient's clinical features (Additional file 5: Figure S5). To further validate the clinical independence of risk score, we performed clinical stratification. Patients in TCGA cohort were divided into different subgroups based on their clinical characteristics (including T stage (T2 and T3–4), N stage (N0 and N1) and Gleason score (Gleason score ≤ 7 and Gleason score > 7), and K-M survival analysis was performed on each subgroup according to the grouping of high and low scores. The results indicated that PFS rates of patients in the high-score group were significantly lower than those in the low-score group in all subgroups (Fig. 7A–F). Patients in GSE70769 cohort were divided into different subgroups based on their clinical characteristics (including T stage (T1 and T2–3), PSA value (PSA ≤ 10 and PSA > 10) and Gleason score (≤ 7 and > 7). The results of K-M survival analysis showed that signature could be successfully applied to patients with T1 and T2–3, PSA ≤ 10, Gleason score ≤ 7, and Gleason score > 7 (Fig. 7G–L). In addition, we compared the risk score differences among the different T, N, and Gleason score subgroups in the TCGA cohort, and the results showed that the risk score in the T3–4, N1, Gleason score > 7 subgroups was significantly higher than that in the T2, N0, and Gleason score ≤ 7 subgroups, These results indicated that risk score can significantly affect the invasion of prostate cancer cells, lymph node metastasis and pathological grade (Additional file 6: Figure S6).

### Functional analyses based on risk signature

**Fig. 8:**
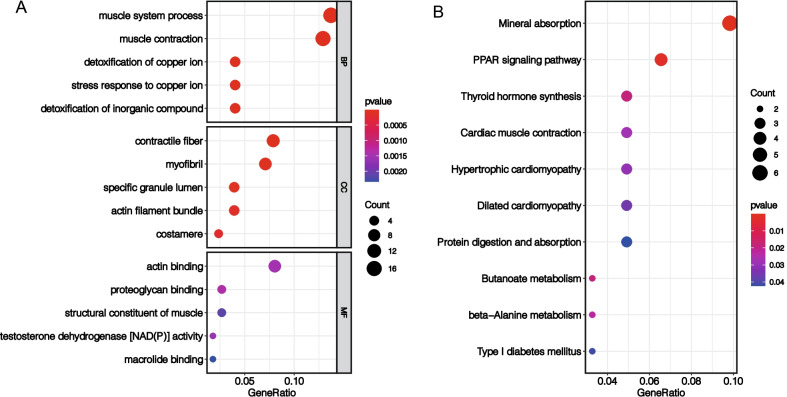
Functional analyses based on risk signature. Gene Ontology (GO) enrichment analysis (**A**) and Kyoto Encyclopedia of Genes and Genomes (KEGG) pathway analysis (**B**) were then performed based on these DEGs. The results reveal that these DEGs are mainly enriched in muscle system process, contractile fiber, actin binding, and PPAR signaling pathway

To further explore differences in gene function and pathway between subgroups by risk score, we used R-package "limma" to obtain DEGs based on a filter criterion of FDR < 0.05 and |log2FC|≥ 0.585. A total of 134 DEGs were identified between high- and low-score groups in TCGA cohort (Additional file 15: Table S9). GO enrichment analysis and KEGG pathway analysis were then performed based on these DEGs. The results revealed that these DEGs were mainly enriched in the muscle system process, contractile fiber, actin binding, and PPAR signaling pathway (Fig. 8A, B).

### High risk score indicates suppressed immune activity

**Fig. 9:**
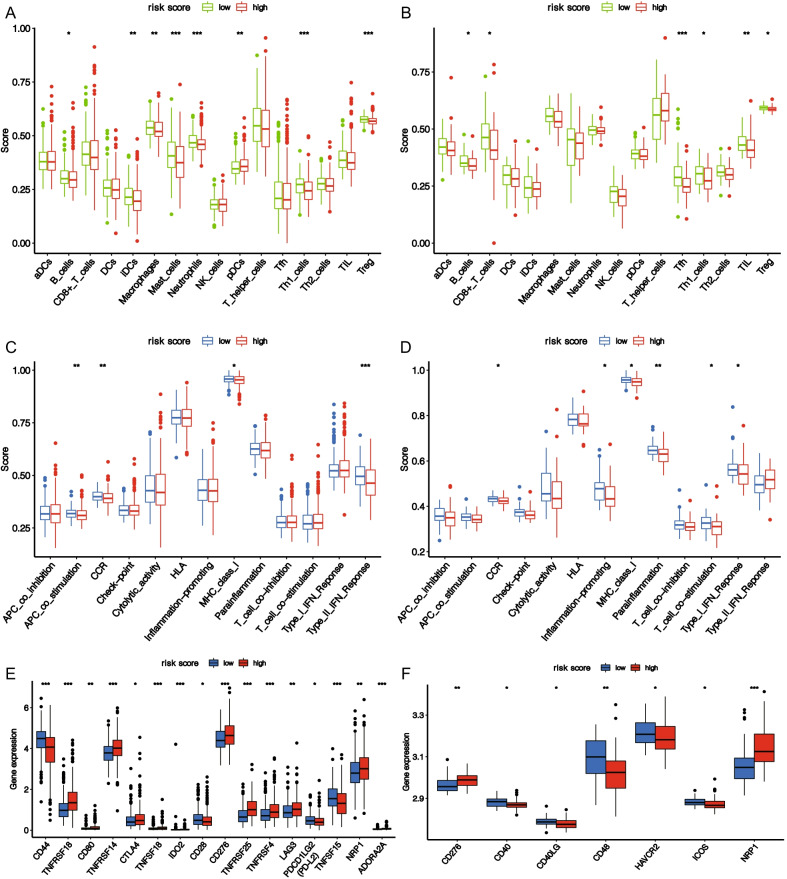
Comparison of ssGSEA scores for immune cells and pathways. Enrichment scores for 16 immunocytes were compared between low- and high-score groups in TCGA cohort (**A**) and GSE70769 cohort (**B**). Enrichment score comparisons for 13 immune-related pathways between low- and high-score groups in TCGA cohort (**C**) and GSE70769 cohort (**D**). Expression comparisons at common immune checkpoints between high- and low-score groups in TCGA cohort (**E**) and GSE70769 cohort (**F**). (Wilcoxon test, *P < 0.05; **P < 0.01; ***P < 0.001.)

Based on functional analysis, we further compared the enrichment scores of 16 immune cells and the activities of 13 immune-related pathways between low- and high-score groups in TCGA and GEO cohorts using ssGSEA (Wilcoxon test, *P < 0.05; **P < 0.01; ***P < 0.001). In TCGA cohort, most immunocytes were higher in low-score group, notably mast cells, neutrophils, th1 cells, and Treg (Fig. 9A). Moreover, the activities of most immune pathways in the low-score group were higher than those in the high-score group, such as APC co-stimulation, Type II IFN response, and MHC class I (Fig. 9C). Similar findings were observed in GEO cohort, where most immunocytes and immune pathways were higher in the low-score group (Fig. 9B, D). The above results indicated that increasing the risk score of tumor cells may inhibit their immunologic activity. The results of comparative analysis of immune checkpoints of different risk score are depicted in Fig. 9E, F. It could be seen that the expression levels of many immune checkpoints, such as CTLA4 and PDCD1LG2 (PD-L2), differed between high and low score groups (Wilcoxon test, *P < 0.05; **P < 0.01; ***P < 0.001).

### Establishment and validation of a nomogram model

**Fig. 10:**
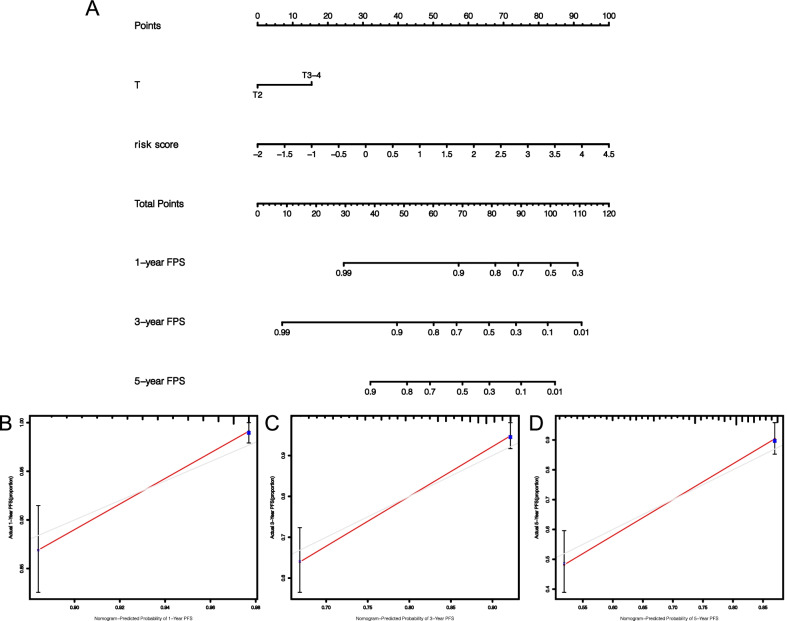
Establishment of nomogram and its performance verification. Nomogram (**A**) combined with age, T stage, and risk score and its calibration diagrams of 1-year (**B**), 3-year (**C**), and 5-year (**D**)

To improve the clinical power of risk signature, we established a nomogram model comprising T stage, and risk score using TCGA set, and then generated calibration charts for 1-, 3- and 5-year periods to demonstrate its accuracy (Fig. 10A–D). Calibration charts revealed that nomogram model was highly accurate, affirming its practicability in predicting prognosis of patients.

## Discussion

Pyroptosis is an inflammation-mediated, programmed cell death [28]. It can not only inhibit the occurrence and development of tumors but also develop a microenvironment that provides nutrition for cancer and accelerates its growth [11]. However, the role of PRGs in prostate cancer (PCa) remains unknown, and we sought to elucidate this role.

PCa is a common malignant tumor found in elderly men worldwide [1]. Biochemical recurrence (BCR) was defined as the second elevation of PSA concentration above 0.2 μg/L, confirmed by two consecutive elevations. It is a determining risk factor for distant metastasis, prostate cancer specificity, and overall mortality [2]. There is evidence that approximately 30% of patients with BCR develop distant metastases with clinical presentation, and 19–27% of patients may die of prostate cancer within ten years without receiving second treatment [29, 30]. Therefore, stratifying patients with post-RP localized PCa into high-risk BCR patients is highly desirable, which may provide more frequent monitoring, early intervention, and even decision-making regarding adjuvant therapy.

It is an effective method to classify samples based on a predetermined gene expression signature [31]. Our classification strategy is based on this approach and classifies PCa based on 52 PRGs expression patterns. We found that the expression of these PRGs was completely different between the two clusters due to heterogeneity. Besides, the prognosis of different clusters varies significantly. Several agreements emerged from our analysis: (1) The expression level of most PRGs was higher in cluster 2; (2) Most DEG expression levels among different clusters were higher in cluster 2; (3) Cluster 1, as a separate subtype, has a worse prognosis; (4) A combination of clinical information and RNA transcriptome data is more likely to reflect cell phenotype. After quantifying immune cells of different clusters, we discovered that many of them had a higher content of cluster 1, particularly T regulatory cells (Tregs). Tregs are the key barrier for tumor immunotherapy, as they actively mediate autoimmune tolerance [32, 33]. In recent years, as medicine has advanced, immune checkpoint inhibitors have become the key treatment measures for many malignant tumors [34]. PD-1, PD-L1, PD-L2, and CTLA4 are common immune checkpoints, where PD-L1 and PD-L2 are the two ligands of PD-1 and belong to B7 family [35, 36]. PD-L1 is widely expressed throughout the body, particularly in immune and cancer cells, whereas PD-L2 expression is relatively limited to professional antigen-presenting cells and increases in response to congenital receptor signals [37]. In addition, CTLA4 antibodies have been demonstrated to reverse T-cell allergy, leading to an antitumor response [38]. We quantified immune checkpoints for different clusters and found cluster 2 had higher levels of these immune checkpoints, indicating that patients with cluster 2 were more likely to benefit from immunotherapy.

Clinical trials have tested anti-tumor molecular targeting drugs in all PCa subtypes, regardless of the underlying molecular subtypes. For instance, immune checkpoint molecules are expressed differently in different subtypes, and immunotherapy should be distinguished accordingly. To enhance clinical utility, we developed a scoring model (risk signature) to quantify prognostic risk based on two clusters. This study provided strong evidence for clinical management of PCa. First, risk score considers the heterogeneity of patients, and PCA results indicate that scoring models can significantly distinguish patients from different risk subgroups. Second, the score can be associated with prognosis. Specifically, risk score characterizes and assigns different weights to both tumor suppressor and tumor promoter genes. The signature included eight genes: CENPA, LCN2, COL7A1, ALB, UBXN10, SPZ1, SCNN1A, and TFF3. The coefficients of UBXN10, SCNN1A, LCN2, and TFF3 are negative, indicating that they can be used as protective factors for patients. Increased expression of these genes improves the prognosis of patients. The coefficients of ALB, SPZ1, CENPA, and COL7A1 are positive, indicating that increased expression increases the risk of poor prognosis in patients. After a careful review of relevant literature, we found that these genes were strongly associated with the occurrence and development of inflammation or malignant tumor. For instance, Masayuki Watanabe stated that Transcription factor SPZ1 might promote TWIST-mediated epithelial-mesenchymal transition in thoracic malignancies [39]. By targeting SLPI, Xu et al. found that LCN2 mediated by IL-17 affects proliferation, migration, invasion, and cell cycle of gastric cancer cells [40]. Sebastiano et al. found that Human COL7A1-corrected induced pluripotent stem cells can be used to treat recessive dystrophic epidermolysis bullosa [41]. Lin et al. found that TFF3 contributes to epithelial-mesenchymal transition in papillary thyroid carcinoma cells via MAPK/ERK signaling pathway [42]. Maibritt et al. found that highly significant and frequent hypomethylation of cancer-specific promoter of TFF3 in malignant prostate cancer [43]. Anjan et al. found that found that overexpression of CENPA was crucial for the growth of prostate cancer [44]. However, we have not found any reports regarding the role of these genes in mediating tumor cell pyroptosis. Third, risk score can significantly distinguish the clinical characteristics of different patients, indicating that as the score increases, the proportion of PCa patients with T3, T4, and N1 increases significantly. Risk score predicts PFS and, to a certain extent, the OS rate. Fourth, data on immune cell infiltration indicate that risk score has significant immunotherapeutic value. The results of ssGSEA reveal that the content of most immune cells in the low score group is higher than that in the high score group, which results in an overactive immune system that may responds better to immunotherapy. There are significant differences in the content of immune checkpoints among different subgroups, which can provide important information for future research on immune checkpoints associated with PCa, particularly PD-L2 and CTLA4. Finally, Risk score focuses directly on PCa cell death patterns compared to other models. Researchers have studied prognosis models of PCa with modification conditions such as ferroptosis [25], m6A [26], and immune score [27]. The higher AUC values in our model can be found by plotting the ROC curve, which means that the accuracy of our risk signature is better than other prognostic models. In addition, to improve the clinical value of risk signature, we established a nomogram model, a score was matched for each variable in the nomogram scoring system. The total score was obtained by summing the scores of all variables of each sample [45]. With the nomogram scoring system, we can predict the PFS possibilities for the corresponding patient in 1, 3, and 5 years, so that risk signature can be more closely combined with clinical applications.

In our study, pyroptosis-related genes are used as the starting point, PCa patients are divided into different subtypes, DEGs are identified, and a prognosis model is constructed that can accurately predict tumor PFS rate of patients. At present, research progress on pyroptosis is limited, and the relationship between prostate cancer and pyroptosis has not been studied. Although we explored the relationship between pyroptosis and prostate cancer to some extent, built and verified a prognosis model from multiple perspectives and different databases, this research still has certain limitations. First, although we have established a risk signature based on PRG clusters, the relationship between its members and pyroptosis has not been reported before, so further in vitro and in vivo experiments are needed to verify the regulatory relationship of these genes on pyroptosis. Second, the bulk expression data we used were enough DNA from a large number of cells to be sequenced, so the sequencing results are a global characterization of these cells. However, due to cellular heterogeneity, the genetic information of cells with the same phenotype can vary significantly, and much of the low abundance information is lost in the overall characterization. To compensate for the limitations of traditional high-throughput sequencing, single-cell sequencing technology comes of age and will play an important role in future studies.

## Conclusion

In summary, the results of this study indicated that pyroptosis was strongly associated with PCa. This study provided a new genetic marker for predicting the prognosis of PCa patients and laid the groundwork for future research on the relationship between pyroptosis-related genes and PCa immunity.

## Supplementary Information

**Additional file 1: Figure S1.** Protein-protein interaction network (PPI) (**A**) of 35 PRGs and their hub genes (**B**).

**Additional file 2: Figure S2.** (**A**) Histogram of immune cell infiltration for different clusters based on the "CIBERSORT" algorithm. Immune cell score (**B**), stromal cell score (**C**), and composite score (**D**) for tumor purity of the two clusters based on "ESTIMATE" algorithm (Wilcoxon test, *p* < 0.001).

**Additional file 3: Figure S3.** Content of immune checkpoints PD-1, PD-L1, PD-L2 and CTLA4 in different clusters (Wilcoxon test, *P < 0.05; **P < 0.01; ***P < 0.001).

**Additional file 4: Figure S4.** Forest map of 110 prognosis-related DEGs obtained by univariate Cox analysis.

**Additional file 5: Figure S5.** Heatmap of eight risk genes with clinical features (chi-square test, *P < 0.05; **P < 0.01; ***P < 0.001).

**Additional file 6: Figure S6.** Risk score boxplot for T, N, and Gleason score subgroups based on TCGA cohort.

**Additional file 9: Table S3.** Two different clusters were identified based on the expression levels of 52 PRGs.

**Additional file 9: Table S3.** Two different clusters were identified based on the expression levels of 52 PRGs.

**Additional file 9: Table S3.** Two different clusters were identified based on the expression levels of 52 PRGs.

**Additional file 10: Table S4.** GSVA analysis results between different clusters (|logFC| > 0.2, adjusted *p* < 0.05).

**Additional file 11: Table S5.** 516 differentially expressed genes (DEGs) between two clusters (|log2FC| > 1 and FDR < 0.05).

**Additional file 12: Table S6.** Prognosis-related DEGs by univariate cox analysis (*p* < 0.05).

**Additional file 13: Table S7.** High- and low-score groups based on risk score in TCGA cohort.

**Additional file 14: Table S8.** High- and low-score groups based on risk score in GEO cohort.

**Additional file 15: Table S9.** 134 DEGs were identified between high and low scoring groups in TCGA cohort (FDR < 0.05 and |log2FC| > 0.585).

## Data Availability

The datasets generated and analysed during the current study are available in the TCGA repository, (https://portal.gdc.cancer.gov) and GEO repository, (https://www.ncbi.nlm.nih.gov/geo/).

## Abbreviations

PCa: Prostate cancer
TCGA: The cancer genome atlas
GEO: Gene expression omnibus
BCR: Biochemical recurrence
K–M: Kaplan–Meier
ROC: Receiver operating characteristics
PRG: Pyroptosis-related gene
PFS: Progression-free survival
RP: Radical prostatectomy
PSA: Prostate-specific antigen
CNV: Copy number variation
PPI: Protein–protein interaction
GSVA: Gene set variation analysis
DEG: Differentially expressed gene
PCA: Principal component analysis
HR: Hazard ratio
CI: Confidence interval
GO: Gene ontology
KEGG: Kyoto encyclopedia of genes and genomes
