# Supplementary figures and images for: A novel defined risk signature based on pyroptosis-related genes can predict the prognosis of prostate cancer
Source: BMC Med Genomics. 2022 Feb 8;15:24. doi: 10.1186/s12920-022-01172-5 (PMC8822680; doi:10.1186/s12920-022-01172-5)

A

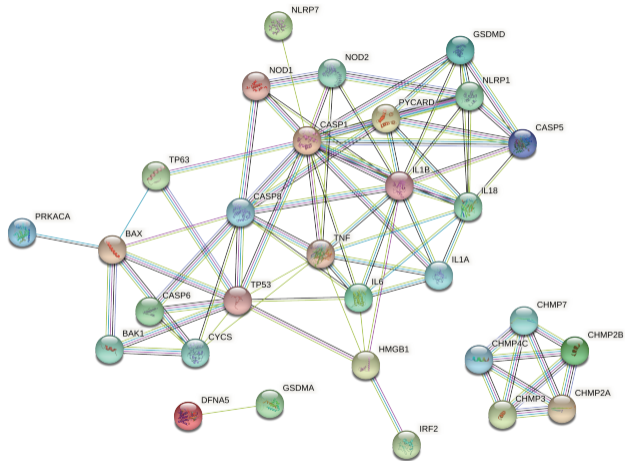

B

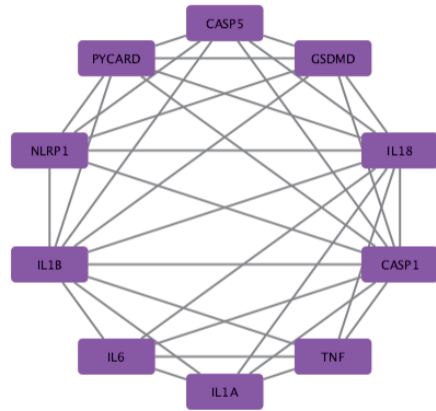

Supplement: Supplementary file 1 — Additional file 1: Figure S1. Protein-protein interaction network (PPI) (A) of 35 PRGs and their hub genes (B). [file 12920_2022_1172_MOESM1_ESM.pdf]

A

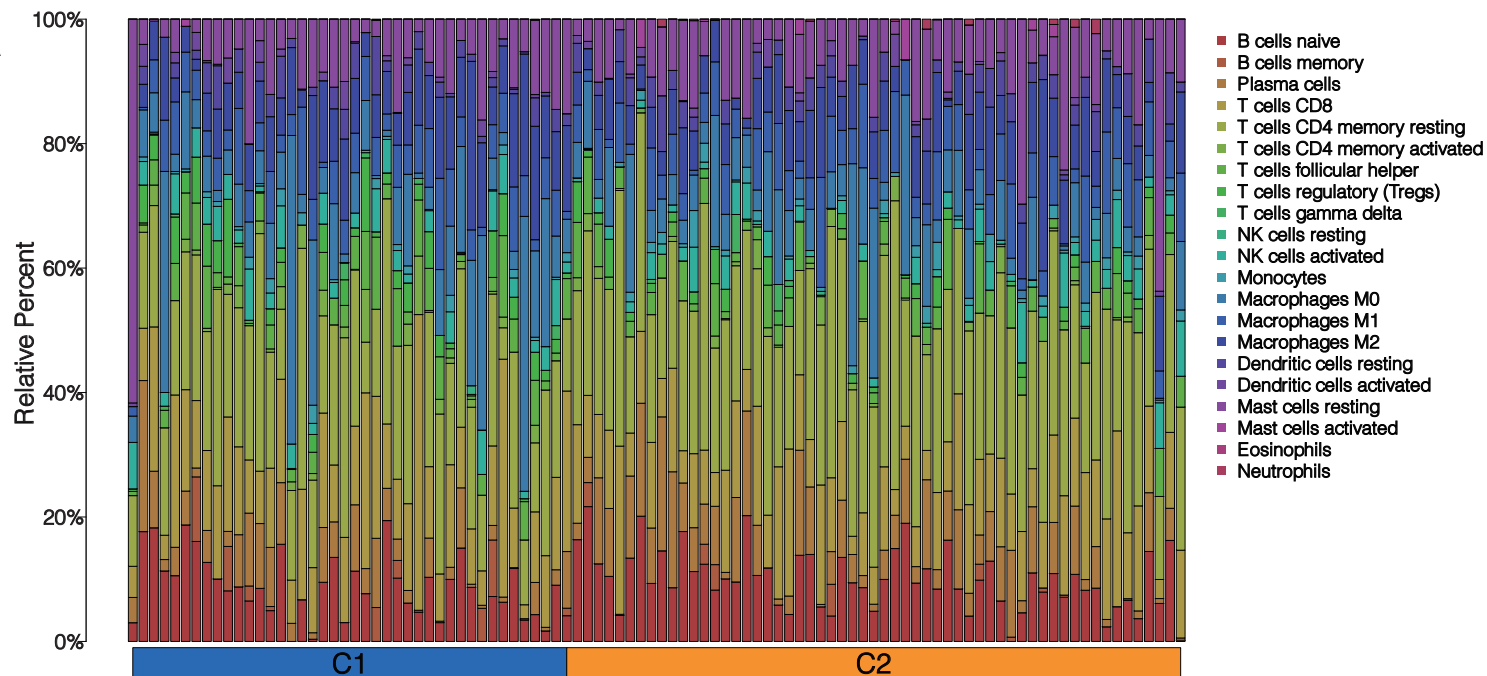

B

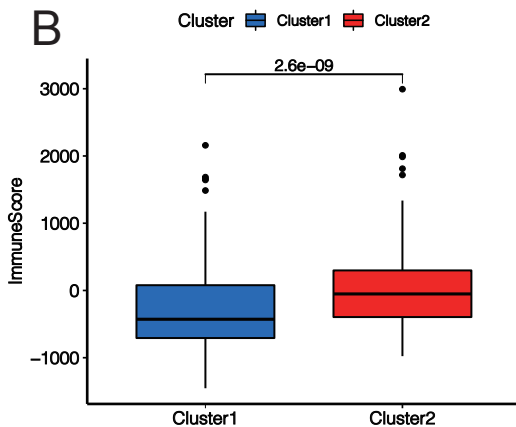

C

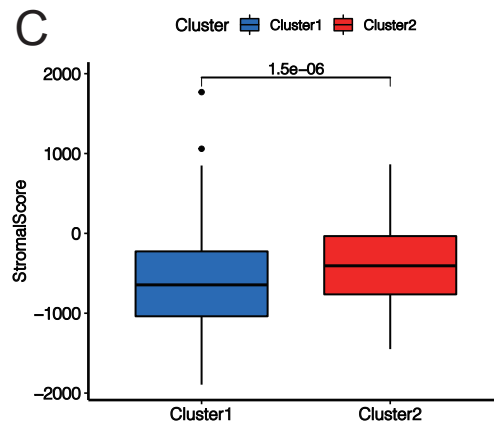

D

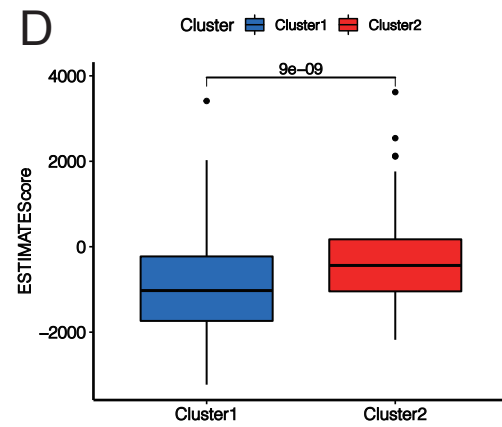

Supplement: Supplementary file 2 — Additional file 2: Figure S2. (A) Histogram of immune cell infiltration for different clusters based on the "CIBERSORT" algorithm. Immune cell score (B), stromal cell score (C), and composite score (D) for tumor purity of the two clusters based on "ESTIMATE" algorithm (Wilcoxon test, p < 0.001). [file 12920_2022_1172_MOESM2_ESM.pdf]

Type C1 C2

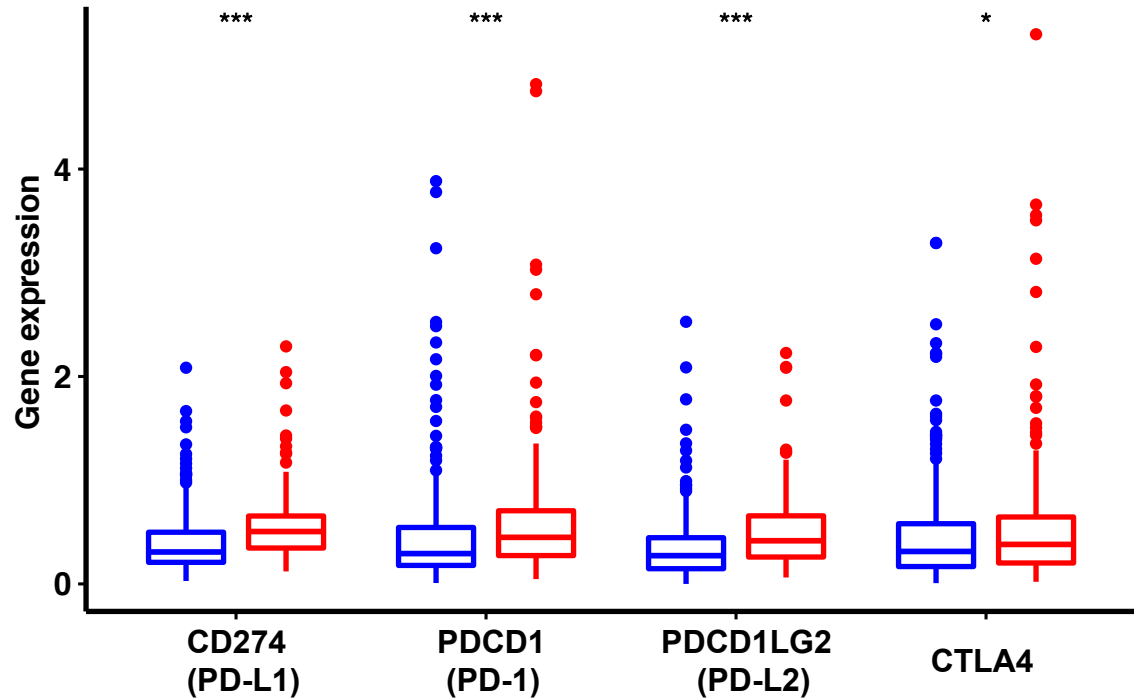

Supplement: Supplementary file 3 — Additional file 3: Figure S3. Content of immune checkpoints PD-1, PD-L1, PD-L2 and CTLA4 in different clusters (Wilcoxon test, *P < 0.05; **P < 0.01; ***P < 0.001). [file 12920_2022_1172_MOESM3_ESM.pdf]

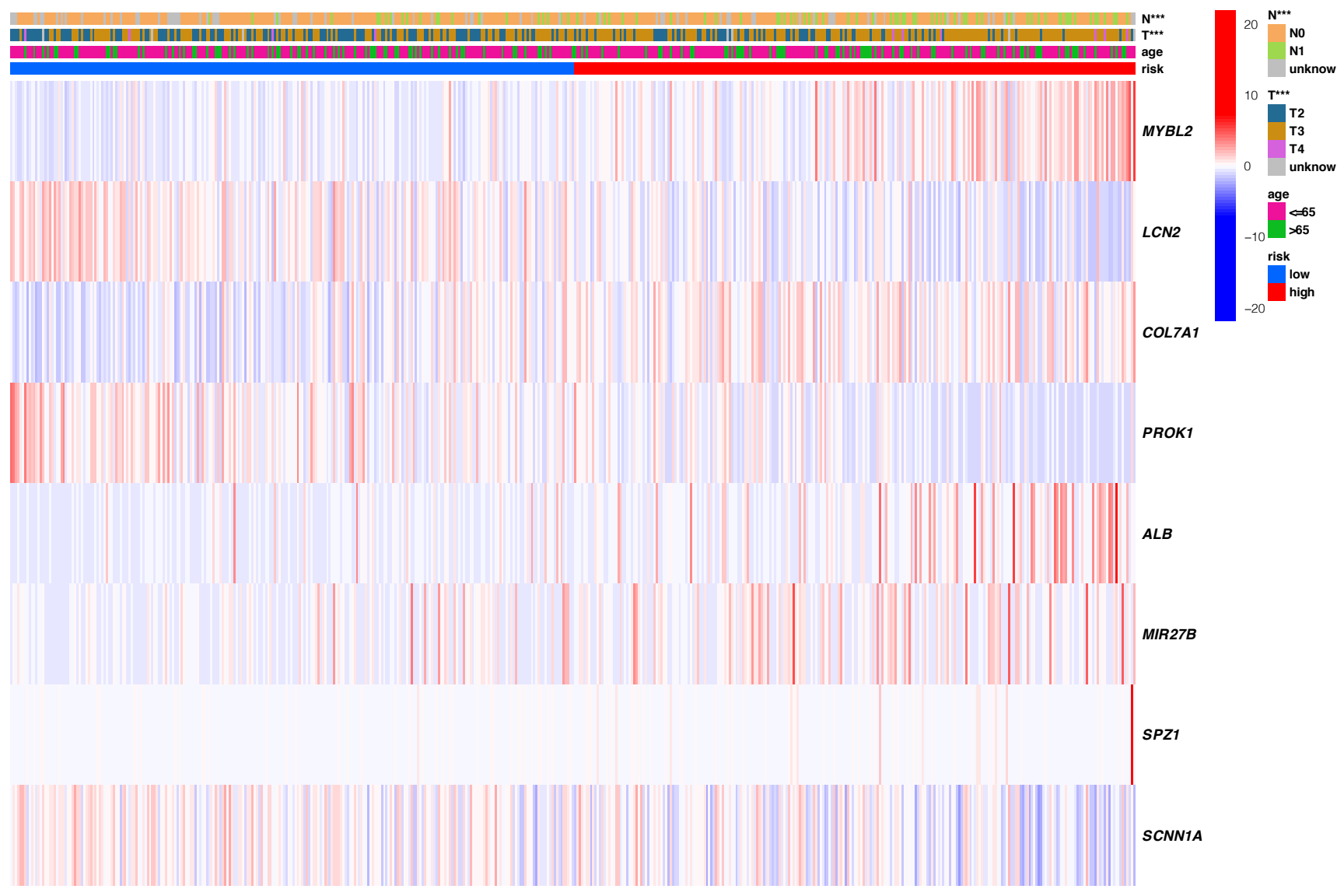

Supplement: Supplementary file 5 — Additional file 5: Figure S5. Heatmap of eight risk genes with clinical features (chi-square test, *P < 0.05; **P < 0.01; ***P < 0.001). [file 12920_2022_1172_MOESM5_ESM.pdf]

A

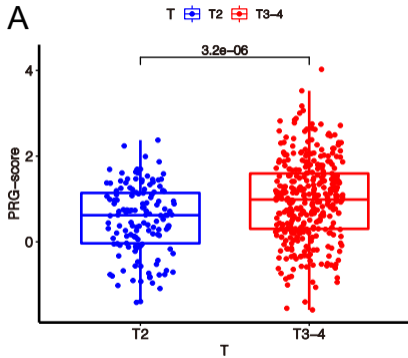

B

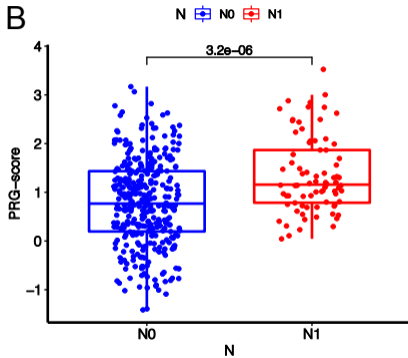

C

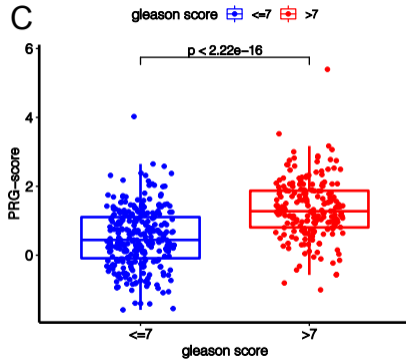

Supplement: Supplementary file 6 — Additional file 6: Figure S6. Risk score boxplot for T, N, and Gleason score subgroups based on TCGA cohort. [file 12920_2022_1172_MOESM6_ESM.pdf]
