# Supplementary material for: A novel defined risk signature based on pyroptosis-related genes can predict the prognosis of prostate cancer
Source: BMC Med Genomics. 2022 Feb 8;15:24. doi: 10.1186/s12920-022-01172-5 (PMC8822680; doi:10.1186/s12920-022-01172-5)

	pvalue	Hazard ratio
EYA4	<0.001	0.310(0.155−0.622)
TP63	0.049	0.811(0.659−0.999)
CENPA	<0.001	2.452(1.814−3.314)
FRMD6	0.005	0.650(0.482−0.877)
FAM124A	0.018	0.513(0.295−0.892)
SLC15A1	0.004	0.467(0.280−0.779)
MYBL2	<0.001	1.850(1.492−2.295)
MSMB	<0.001	0.842(0.767−0.923)
GNG13	0.023	1.309(1.038−1.651)
CBX2	<0.001	2.034(1.511−2.738)
COL4A6	0.006	0.614(0.435−0.867)
SPON1	0.023	0.750(0.586−0.961)
CHRDL1	0.001	0.758(0.641−0.896)
LCN2	<0.001	0.825(0.740−0.919)
PENK	0.032	0.683(0.481−0.969)
PCDH7	0.008	0.626(0.443−0.883)
ANO5	0.001	0.450(0.277−0.731)
EDN3	0.001	0.442(0.267−0.732)
CPNE7	0.032	1.226(1.018−1.477)
COL27A1	0.040	1.458(1.018−2.088)
SYNPO2	0.001	0.760(0.642−0.899)
FAT4	0.043	0.570(0.330−0.982)
COL7A1	0.002	1.463(1.153−1.855)
TNS1	0.009	0.771(0.634−0.938)
ARHGDIG	<0.001	1.515(1.227−1.870)
MAML2	0.011	0.645(0.459−0.906)
SRD5A2	<0.001	0.587(0.464−0.743)
RND2	0.014	0.502(0.289−0.871)
LTF	0.050	0.926(0.858−1.000)
CD177	0.010	0.867(0.778−0.966)
KCTD14	0.003	0.569(0.393−0.824)
FBXO17	0.039	0.668(0.456−0.979)
COL17A1	0.046	0.811(0.661−0.996)
ZNF750	0.031	0.739(0.562−0.972)
ALDH1A2	<0.001	0.564(0.410−0.775)
NEK2	<0.001	2.062(1.581−2.689)
ROR2	0.048	0.765(0.587−0.998)
SPATA18	0.006	0.593(0.410−0.858)
PROK1	<0.001	0.588(0.453−0.763)
P2RX1	0.019	0.678(0.489−0.939)
APOBEC3C	0.034	0.784(0.627−0.982)
KIAA1210	0.004	0.635(0.467−0.862)
ATP2B4	0.004	0.716(0.570−0.899)
BIRC5	<0.001	1.990(1.576−2.515)
FAM107A	<0.001	0.635(0.503−0.800)
COL23A1	0.013	0.560(0.355−0.885)
MNX1	0.030	1.394(1.032−1.884)
ALB	<0.001	1.286(1.118−1.479)
ACSS3	0.010	0.423(0.219−0.817)
MPP2	0.032	0.611(0.390−0.958)
OLFM4	0.015	0.888(0.807−0.978)
GGT6	0.022	0.741(0.573−0.958)
MR1	0.041	0.664(0.448−0.984)
ITGA2	0.018	0.687(0.502−0.939)
ARHGAP20	0.011	0.448(0.241−0.835)
MYH11	<0.001	0.783(0.685−0.896)
ANGPT1	0.001	0.568(0.405−0.797)
CCK	0.028	0.806(0.665−0.977)
ACOX2	0.004	0.590(0.413−0.844)
ATCAY	0.007	0.500(0.302−0.828)
MEIS2	0.025	0.702(0.515−0.957)
NEFH	0.039	0.916(0.842−0.996)
SERPINB5	0.026	0.717(0.535−0.961)
UBXN10	<0.001	0.280(0.152−0.515)
SLC8A1	0.017	0.591(0.384−0.911)
GNAL	0.007	0.534(0.339−0.843)
PRIMA1	0.026	0.656(0.453−0.951)
UBE2C	<0.001	1.770(1.460−2.146)
MALAT1	0.009	1.270(1.060−1.522)
ZNF185	<0.001	0.648(0.502−0.836)
SLC18A2	0.018	0.483(0.264−0.884)
SLC26A4	0.006	0.767(0.634−0.926)
MIR27B	0.033	1.340(1.023−1.755)
CRIP3	0.038	0.509(0.268−0.964)
PDZRN4	0.002	0.597(0.429−0.831)
SLC2A5	0.021	0.721(0.545−0.952)
SPZ1	0.007	1.411(1.097−1.816)
PGM5	<0.001	0.719(0.606−0.853)
HES6	0.010	1.302(1.064−1.594)
STAC	0.003	0.569(0.395−0.821)
JPH4	<0.001	0.585(0.434−0.787)
SORBS1	0.005	0.755(0.620−0.919)
AMH	<0.001	1.889(1.514−2.356)
WFDC2	0.012	0.845(0.741−0.963)
MYOCD	<0.001	0.543(0.391−0.754)
SCNN1A	0.018	0.806(0.674−0.964)
MMP11	<0.001	1.547(1.287−1.859)
TFF3	0.037	0.906(0.825−0.994)
C2orf88	<0.001	0.345(0.186−0.640)
FOXI1	0.022	0.572(0.355−0.923)
CLIC6	0.010	0.677(0.503−0.911)
SYNM	<0.001	0.748(0.633−0.884)
B3GALT2	<0.001	0.331(0.192−0.569)
SNAI2	0.023	0.759(0.598−0.962)
TROAP	<0.001	2.842(2.088−3.867)
IP6K3	<0.001	0.410(0.252−0.667)
TRPC4	0.015	0.568(0.360−0.897)
MYLK	0.001	0.757(0.638−0.897)
AOX1	0.001	0.659(0.510−0.850)
FLNC	0.002	0.777(0.662−0.911)
HPDL	0.006	0.399(0.206−0.773)
GLIS1	0.047	0.667(0.447−0.996)
FGF10	0.014	0.507(0.295−0.871)
CA14	<0.001	0.280(0.134−0.587)
ZNF516	0.022	0.655(0.456−0.940)
AHNAK2	0.022	0.644(0.443−0.937)
PGR	0.029	0.501(0.269−0.933)
GPX3	0.023	0.808(0.672−0.971)
ADRA1A	0.003	0.416(0.233−0.741)
FLNA	0.009	0.793(0.667−0.944)

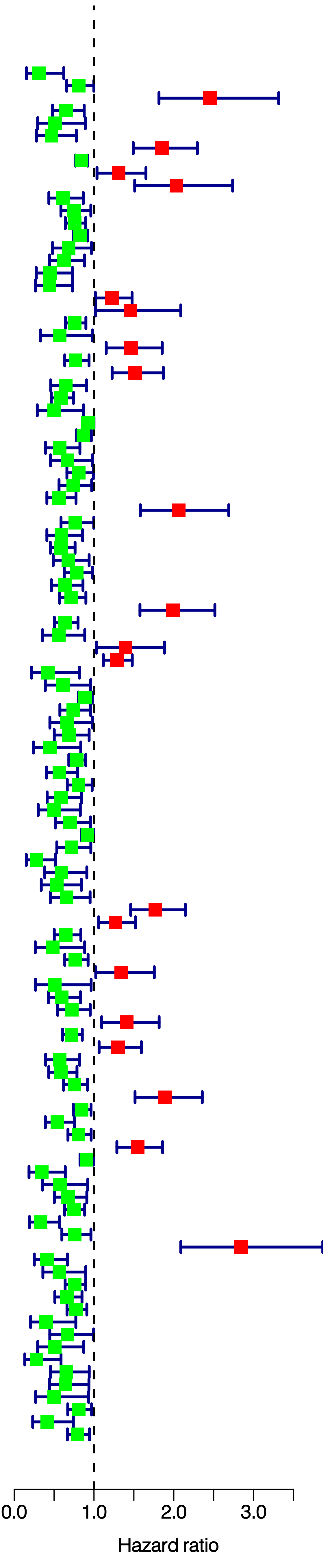

Supplement: Supplementary file 4 — Additional file 4: Figure S4. Forest map of 110 prognosis-related DEGs obtained by univariate Cox analysis. [file 12920_2022_1172_MOESM4_ESM.pdf]
